# Effect of Neck-Deep Immersion in Cool or Thermoneutral Water on Blood Glucose Levels in Individuals With Type 1 Diabetes

**DOI:** 10.1210/jendso/bvad128

**Published:** 2023-10-18

**Authors:** Kristina J Abramoff, Lauren L De Souza, Shane K Maloney, Elizabeth A Davis, Timothy W Jones, Paul A Fournier

**Affiliations:** Department of Sport Science, Exercise and Health, School of Human Sciences, The University of Western Australia, Crawley, WA 6009, Australia; Department of Anatomy Physiology and Human Biology, School of Human Sciences, The University of Western Australia, Crawley, WA 6009, Australia; Children's Diabetes Centre, Telethon Kids Institute, Nedlands, WA 6009, Australia; Department of Sport Science, Exercise and Health, School of Human Sciences, The University of Western Australia, Crawley, WA 6009, Australia; Department of Anatomy Physiology and Human Biology, School of Human Sciences, The University of Western Australia, Crawley, WA 6009, Australia; Children's Diabetes Centre, Telethon Kids Institute, Nedlands, WA 6009, Australia; Children's Diabetes Centre, Telethon Kids Institute, Nedlands, WA 6009, Australia; Department of Sport Science, Exercise and Health, School of Human Sciences, The University of Western Australia, Crawley, WA 6009, Australia; Children's Diabetes Centre, Telethon Kids Institute, Nedlands, WA 6009, Australia

**Keywords:** water immersion, hypoglycemia, cool water immersion, thermoneutral water immersion, type 1 diabetes mellitus, blood glucose

## Abstract

**Context:**

It is unclear whether immersion in cool water, typical of many beaches, increases the concentration of blood glucose in individuals with type 1 diabetes mellitus (T1DM).

**Objective:**

To test the hypothesis in individuals with T1DM that immersion neck-deep in cool water (COOL) causes an increase in blood glucose concentration, but not exposure to thermoneutral water (THERMO) or thermoneutral air.

**Methods:**

Eight overnight-fasted participants with T1DM were exposed for 60 minutes on separate days to 3 experimental conditions: cool water (COOL, 23 °C); thermoneutral water (THERMO, 33.5 °C); or thermoneutral air (24 °C). They then recovered for 60 minutes on land at 24 °C. At time intervals, we measured: blood glucose and plasma insulin concentration, rate of carbohydrate and fat oxidation, skin and core temperature, subcutaneous blood flow, and shivering via electromyography.

**Results:**

There was no change in blood glucose concentration during the 3 experimental conditions (*P* > .05). During recovery after COOL, blood glucose increased (*P* < .05) but did not change in the other 2 conditions. The rate of carbohydrate oxidation during and early after COOL was higher than in the other 2 conditions (*P* < .05), and COOL led to a decrease in subcutaneous blood flow and the concentration of plasma insulin (*P* < .05).

**Conclusion:**

Cool or thermoneutral neck-deep immersion in water does not cause a change in the concentration of blood glucose in people with T1DM, but on-land recovery from COOL causes an increase in blood glucose that may be due, at least in part, to the accompanying decrease in plasma insulin.

Many factors, such as physical activity and relative hyperinsulinemia, can increase the risk of hypoglycemia in individuals with type 1 diabetes mellitus (T1DM) [1-3]. For this reason, many guidelines have been designed to help people with T1DM adjust their insulin dose and carbohydrate (CHO) intake to reduce their risk of hypoglycemia [1, 2, 4]. Unfortunately, one limitation shared by these guidelines is that they provide little information on the impact that some environmental conditions may have on the risk of hypoglycemia. Immersion in cool/cold water that is typically found at many beaches such as in Australia and many other countries [5], is a potential environmental factor that may, in theory, increase the risk of hypoglycemia. That this is a risk factor is suggested indirectly by the observation that exposure to cold air or cold water increases the resting metabolic rate of people without diabetes to levels equivalent to those associated with low to moderate intensity exercise (∼20-46% of V̇O_2peak_) [6-10]. Also, mild cold exposure in people without diabetes can increase their rates of plasma glucose oxidation by 2.1-fold [7], with plasma glucose being an important fuel that supports cold-induced shivering [11]. Consistent with the observation that the rate of glucose oxidation increases during cold exposure, some, but not all studies [7, 8], have reported that immersion in cold water (18 °C) or exposure to cold air (10 °C) is associated with a small decrease in the concentration of plasma glucose in individuals without diabetes [9-12]. On the basis of these latter studies [9, 12], immersion in cold water could be expected to increase the risk of hypoglycemia in individuals with T1DM.

However, one factor likely to oppose the effect of cold water on the risk of hypoglycemia in insulin-treated people with T1DM is the decrease in peripheral blood flow that normally accompanies cold exposure [13, 14]. Since the rate of insulin absorption is highly dependent on subcutaneous blood flow [15] and skin temperature [16], the decrease in skin temperature and peripheral blood flow that accompanies cold exposure [13, 14, 17] could be expected to decrease the absorption rate of subcutaneous insulin in cold-exposed insulin-treated individuals with T1DM. In support of this prediction, Rönnemaa and Koivisto [17] found that the absorption rate of an insulin bolus that was given with a meal was lower in cold compared to warm ambient temperature and was associated with a larger postprandial increase in plasma glucose concentration. These observations suggest that cold exposure could lead to a decrease in the rate of insulin absorption and an increase in the concentration of blood glucose, therefore implying a lower risk of hypoglycemia in insulin-treated individuals with T1DM.

The effect of immersion in cool/cold water per se on the risk of hypoglycemia has never been examined in individuals with T1DM with or without the confounding effect of physical activity. For this reason, the primary objective of this study was to test the hypothesis that standing neck-deep in cool water (23 °C) may be associated with an increase in the concentration of blood glucose but not during standing in thermoneutral water (33.5 °C) or on land (24 °C). This is an important issue to address, given that beachgoing is popular in many countries like Australia (nearly 2 out of 3 Australian adults visited the beach in 2018, and more than 60% of these individuals immersed themselves in water while at the beach) [18], the water at many beaches in these countries is cool or cold (23 °C or less, even during summer) [5], and many individuals move little and avoid swimming while in the water [19] at the beach in part because many find waves as being hazardous [18]. If our hypothesis were to be refuted and immersion in cool water were to cause a rapid decrease in the concentration of blood glucose, such an information could eventually be included in future guidelines to help individuals with T1DM lower their risk of exercise-induced hypoglycemia while at the beach. For this reason, the testing of our hypothesis is timely and may lead to an update to guidelines for hypoglycemia prevention.

## Methods

### Participants

Eight, complication-free adolescents or adults (aged 16-40 years; 3 female, 5 male) whose T1DM was treated with insulin were recruited for the study, (descriptive characteristics in Table 1). All of the participants had to be proficient swimmers with no prior regular cold water exposure or involvement in any cold acclimatization regimen. Exclusion criteria included pregnancy and any medical condition other than T1DM, and all female participants were tested during the follicular phase of their menstrual cycle. We calculated that a sample size of 8 was large enough to detect a significant difference between treatments with a statistical power of 0.8 and an effect size of 1.0 at a significance level of *P* < .05. Written informed assent and consent were obtained from each participant and their parent/guardian if younger than 18 years. Ethics approval was granted by the Human Research Ethics Office at the University of Western Australia (RA-4-20-4563).

**Table 1.** Descriptive characteristics of participants (N = 8)

Characteristics	Mean (SD)/N
Age (years)	29.6 (10.5)
Height (cm)	173.9 (7.9)
Mass (kg)	76.1 (13.8)
Body mass index (kg/m^2^)	25.0 (2.6)
Adipose tissue (%)	23.0 (12.8)
Glycated hemoglobin (mmol/mol)	52.67 (8.01)
Duration of diabetes (years)	12.75 (8.36)
Treatment with multiple daily insulin injections (N)	5
Treatment with insulin pump (N)	3

### Overview of Experimental Design

A randomized, counterbalanced, repeated measure study design was adopted to examine the effect of standing neck-deep in cool or thermoneutral water as well as standing on land while exposed to thermoneutral air on the concentration of blood glucose measured before, during, and after immersion in individuals with T1DM. The participants completed a familiarization session and were exposed on different days to 3 separate testing conditions in the Aquacycle Laboratory and Exercise Physiology Laboratory in the School of Human Sciences at the University of Western Australia. All testing sessions were performed in the morning after an overnight fast, with the participants acting as their own control. Also, the participants were only under the influence of their basal insulin dose, a condition associated with a low risk of hypoglycemia compared with other times during daytime [1, 2, 4]. The order of exposure to the following 3 experimental conditions was randomized for each participant. At time intervals before, during, and after treatment, a number of physiological variables were measured (see below for more details).

### Familiarization Session

On their first visit, all of the participants were familiarized with our research team, equipment, and procedures, including the water immersion protocol. Participants were provided with an opportunity to ask questions and to withdraw their assent/consent. Anthropometric measurements were taken (height and body mass) as well as body composition (% fat mass) using Lunar dual energy x-ray absorptiometry (iDXA, GE Healthcare, Madison, WI, USA). The body fat level and body mass index were measured to exclude obese participants from the study, as excessive adiposity can affect the response of core temperature to exposure to low temperature [20].

### Testing Sessions

Participants prepared for each testing day by completing an exercise/sleep diary and a food diary (details in Table 2). The participants ate meals typical of their normal diet the night before testing, and they were asked to have the same meals prior to each testing session. The matching of meal intake prior to testing is an important precaution as the rates of CHO and fat oxidation at rest are influenced by the diet consumed over the preceding 24 hours [21]. For 2 days before testing, the participants were asked to abstain from vigorous physical activity. Alcohol or caffeine were prohibited on the day prior to testing and on the test day. Participant fasted overnight and ingested ∼500 mL of water the night before testing to ensure morning hydration.

**Table 2.** Sleep quantity and macronutrient intake of the participants on the day prior to testing (N = 8)

Variables	Trials	Mean (SD)	*P* Value
Sleep (hours)	Cool water	7.29 (0.89)	C vs T, *P* = .47
	Thermoneutral water	7.14 (0.75)	C vs L, *P* = .60
	Land	7.42 (0.88)	T vs L, *P* = .39
Carbohydrate (g/day)	Cool water	215 (38)	C vs T, *P* = .93
	Thermoneutral water	224 (52)	C vs L, *P* = .97
	Land	209 (39)	T vs L, *P* = .81
Fat (g/day)	Cool water	94 (17)	C vs T, *P* = .98
	Thermoneutral water	98 (15)	C vs L, *P* = .70
	Land	92 (21)	T vs L, *P* = .82
Protein (g/day)	Cool water	92 (13)	C vs T, *P* = .87
	Thermoneutral water	94 (20)	C vs L, *P* = 1.00
	Land	100 (16)	T vs L, *P* = .85
Total energy (kJ/day)	Cool water	8900 (975)	C vs T, *P* = .99
	Thermoneutral water	8966 (827)	C vs L, *P* = 1.00
	Land	8905 (990)	T vs L, *P* = .99

Abbreviations: C, cool water; L, land; T, thermoneutral water.

Participants arrived at the laboratory while only under the influence of their basal insulin dose. To this end, the 5 participants who were treated using multiple daily insulin injections (MDI) were required to self-administer their slow-acting insulin in the abdomen, either the night and/or the morning of testing, to mark the injection site with a marker pen, and to abstain from injecting any bolus of fast-acting insulin on the morning of testing. The types of basal insulin used by the participants were Levemir (n = 1) and Lantus (n = 4). Participants using insulin pumps (n = 3) were asked to move/keep their insulin infusion site to the abdomen, and to maintain their insulin pump at the basal setting after their last insulin bolus the night prior to testing. The types of insulin administered via the insulin pump included Novorapid (n = 2) and Humalog (n = 1). Each participant kept her/his type, timing, and dosage of basal insulin administration the same prior to each of their 3 experimental sessions. Each participant was tested at the same time in the morning of the 3 experimental sessions to ensure that the time between the administration of basal insulin and time of testing was uniform between the 3 conditions.

On arrival at the laboratory, the concentration of blood glucose was measured, and the testing session proceeded only if the concentration of blood glucose was between 5 and 10 mmol/L. A catheter was then inserted into an antecubital vein for the collection of venous blood samples before and at the end of the immersion, and at 60 minutes post immersion. Of note, one of the participants had a history of syncope during venipuncture and was therefore excluded from catheterization. Before entering the temperature-controlled water tank, each participant was fitted with the appropriate equipment to measure respiratory gases, core and skin temperature, heart rate, cutaneous blood flow, and electromyographic (EMG) activity of the deltoid and trapezius muscles as described in more detail below. Although the use of a water tank does not fully mimic the conditions found at the beach (eg, lack of waves and minimal scope of movement for the participants), it provides an experimental model suitable for the collection of the aforementioned physiological variables under very rigorous and reproducible conditions.

Before entering the water tank, the participants were fitted with a safety harness connected to a ceiling hoist (Ceiling Hoist, Motor- Transactive Xtra fixed, Aidacare Health Equipment, Australia) to allow the experimenter to rapidly remove the participant from the water tank in case of an emergency. The water level was individually adjusted for each participant so that water was level with the sternoclavicular notch. Also, the cannulated arm was allowed to rest on a foam buoy to prevent water contact and to facilitate blood sampling.

The participants were exposed for 1 hour on separate testing days to the following conditions that were administered following a counterbalanced study design:

1. Standing still on land (LAND) at an air temperature of 24 °C to achieve thermal balance without relying on any shivering thermogenesis [22].
2. Standing still in neck-deep, thermoneutral water (THERMO) at 33.5 °C [22]. Of note, thermoneutrality in water is achieved at a higher temperature than in air [22].
3. Standing still in neck-deep cool water (COOL) at 23 °C. Water immersion at 23 °C was chosen because this temperature is (a) below the lower critical water temperature [23]; (b) conducive to shivering, but without causing a major decrease in core temperature during one hour of water immersion [24]; (c) the temperature of the water at many beaches is cool (18-23 °C) [5] ; and (d) is well tolerated by most participants, an important issue as colder water would have made the recruitment of participants even more challenging than it already was for our study.

After each testing session, the participants were dried off and then asked to sit for 1 hour in a room at 24 °C. Our experiment was designed so that the participants were in an overnight fasted basal insulinemic state at the time of testing, a period during daytime when the risk of hypoglycemia at rest or during exercise is low. Therefore, our experimental design and chosen ambient temperature imitates the conditions of beachgoing that can be found before breakfast [25].

Before and every 15 minutes during and after water immersion (or during land standing), the following variables were measured; glucose concentration in capillary blood taken from the fingertips, ventilation rate, and the concentration of expired gases to measure the rate of CHO and fat oxidation, core and skin temperature, heart rate, blood pressure (systolic and diastolic), cutaneous blood flow, and EMG activity of the deltoid and trapezius muscles. All of the relevant protocols to measure those variables are described in detail in the following paragraphs.

### Blood Sampling and Analyses of Glucose and Insulin

To facilitate the sampling of blood and arterialize the composition of the blood samples, one of the arms and hands of the participants were kept out of water by being placed on a foam buoy, as mentioned above, and the fingertip that was targeted for blood sampling was heated using a heated wheat bag (Feel Good Australia, Victoria). Capillary blood glucose from a fingertip was sampled and analyzed using a glucose analyzer (HemoCue® Glucose 201 RT System), as outlined by Dugan and colleagues [26]. To measure the level of plasma insulin, the arm that had been cannulated was heated for 5 minutes as described above (Feel Good Australia, Victoria) prior to the collection of venous blood via the antecubital vein. The process of preparing venous blood for the analysis of bioactive free insulin is described in Dugan and colleagues [26]. Plasma insulin levels were assayed by a local clinical laboratory (PathWest, Osborne Park, West Australia, Australia) using the Architect chemiluminescent microparticle immunoassay system (Abbott Architect i2000SR, Illinois, USA).

### Measurement of Core and Skin Temperature and Heart Rate

Mean skin temperature was measured with 4 thermocouples and data logger (Thermocouple type T, exposed tip, PTFE insulated, Pico Technology, United Kingdom). Mean skin temperature was calculated using the following equation: 0.3 × T chest + 0.3 × T upper arm + 0.2 × T thigh + 0.2 × T gastrocnemius muscle [27]. Core temperature was measured using a Braun Pro 4000 ThermoScan thermometer that measured temperature in the aural canal. Heart rate was measured using a heart rate (HR) monitor (Polar FT, Kempele, Finland).

### Measurement of Subcutaneous Blood Flow

Relative changes in subcutaneous blood flow (skBF) were measured using a laser Doppler flowmeter (LDF; Blood Flow Meter, ADInstruments Pty Ltd, Sydney) and a skin surface probe (OxyFlo Probe, Oxford Optronix Ltd., Oxford, UK) [28]. The probe was secured using an adhesive mounting ring (Adhesive Rings for Probes, ADInstruments Pty Ltd, Sydney), and placed near to the site of insulin injection/infusion at the level of the inferior abdomen to minimize movement artifact and to optimize measurement reproducibility. Measurements of subcutaneous blood flow at the different targeted time points were obtained by averaging 5 minutes of digital recording at the corresponding digital recording time stamp [29].

### Electromyography Protocol

Involuntary muscle contraction, of the type that takes place during shivering thermogenesis, was measured using EMG recordings of the trapezius and deltoid muscles. Surface electrode pads were placed 2 cm apart over the belly of each muscle, and a reference electrode was placed on the ventral surface of the wrist [30]. The EMG electrodes were connected to a data acquisition and signal processing unit system (Power Lab 26 T; LTS), with band pass filters of 0.5-20 Hz and a sampling rate of 1 kHz. Data were exported and later analyzed using Labchart (v7.0, ADInstruments Pty Ltd, Sydney). The measurements of EMG activity at different time points were obtained by taking 200-ms segments of digital recording at the corresponding time stamp [31].

### Collection of Respiratory Gases

Participants wore a mouthpiece for expired gas collection performed every 15 seconds, and ventilation rate was analyzed using indirect calorimetry (TrueOne 2400, Parvo Medica Inc, Utah, USA). Carbohydrate and fat oxidation rates were calculated as described by others [32, 33]. Prior to using the system, appropriate calibration of the oxygen and carbon dioxide sensors were performed using calibration gases. The volume transducer was calibrated by injections of air from a 3-L syringe. Respiratory gases were collected and measured from a 5-minute average at each respective time point.

### Statistical Analyses

Two approaches were adopted to analyze our data, each with its strengths and limitations. Using the Statistical Package for the Social Sciences (SPSS) version 25.0 (IBM, Inc., New York, NY), the area between the variable × time curve and baseline (ABC) was compared between conditions to keep the risk of type 1 error to a minimum. Of note, since baseline values are subtracted from the variable × time curve, when the values on the variable × time curve are higher than baseline values the area derived is positive, but when the values on the variable × time curve are lower than baseline values then the area derived is negative. Significance was set at *P* < .05 for comparisons between ABCs. One limitation of such a conservative, but rigorous ABC-based analysis is that it can fail to reveal very significant (eg, *P* < .001) and important intra-condition differences that only a traditional multiple comparison approach can uncover (eg, temporal changes in the variables measured in this study). For this reason, our results were also analyzed using a two-way analysis of variance (ANOVA) with repeated measures followed by a Fisher least significant difference (LSD) post hoc test, and with significance set at *P* < .05. Cohen d effect sizes were also calculated using SPSS and interpreted in accordance with previous recommendations [34]. With the exception of the descriptive characteristics of the participants that are expressed as mean (SD), all of the results are expressed as mean ± standard error of the mean (SEM).

## Results

### Descriptive Characteristics of the Participants

The descriptive characteristics of the participants are provided in Table 1.

### Matching of Experimental Conditions

On the day prior to each testing day, there was no significant difference in carbohydrate, fat, protein, or total energy intake between conditions (*P* > .05, Table 2). Sleep duration and quality were also well matched between conditions (*P* > .05, Table 2). The ambient temperature and humidity of the recovery room was the same for each of the experimental conditions (*P* > .05, Table 3).

**Table 3.** Environmental characteristics of the recovery condition after each testing session (N = 8)

Variables	Trials	Mean (SD)	*P* Value
Air temperature (°C)	Cool water	23.6 (2.7)	C vs T, *P* = .39
	Thermoneutral water	24.4 (1.5)	C vs L, *P* = .73
	Land	23.8 (1.3)	T vs L, *P* = .23
Relative humidity (%)	Cool water	46.1 (7.3)	C vs T, *P* = .46
	Thermoneutral water	43.6 (7.8)	C vs L, *P* = .97
	Land	46.2 (3.5)	T vs L, *P* = .42

Abbreviations: C, cool water; L, land; T, thermoneutral water.

### Blood Glucose

**Figure 1.**
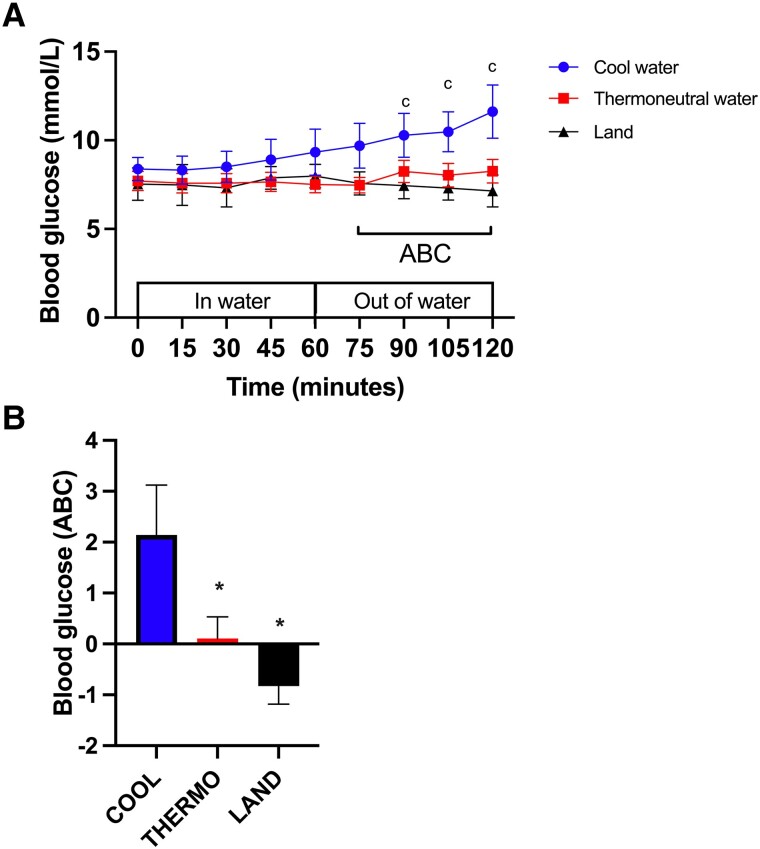
Effect of standing in cool water, thermoneutral water or on land on the concentration of blood glucose (mmol/L) over (A) time and (B) area between the baseline and curve (ABC). (A) c, indicates ignificant difference in the cool water condition compared to baseline. (B) *, Significant difference in area between the baseline and curve (ABC) compared to COOL. Data are displayed as mean ± SEM (N = 8).

At time zero, the concentration of blood glucose did not differ between the conditions (COOL vs THERMO, *P* = .87; COOL vs LAND, *P* = .80; THERMO vs LAND, *P* = .99; Fig. 1). At 60 minutes, there was no significant change in the concentration of blood glucose relative to baseline in any of the 3 experimental conditions (COOL, *P* = .19; THERMO, *P* = .99; LAND, *P* = .95). During recovery (t = 120 minutes), there was no change in the concentration of blood glucose from baseline in LAND (*P* = .14) or THERMO (*P* = .74), but there was a significant increase in the concentration of blood glucose in COOL, with a large effect size from baseline (*P* < .0001, Cohen d = 1.06; Fig. 1). During recovery, the area between the baseline and the concentration of blood glucose vs time curve was significantly more positive in COOL compared to either THERMO or LAND, with large effect sizes between COOL and LAND (*P* = .018, Cohen d = 1.40; Fig. 1) and THERMO (*P* = .02, Cohen d = 0.95).

### Plasma Insulin

**Figure 2.**
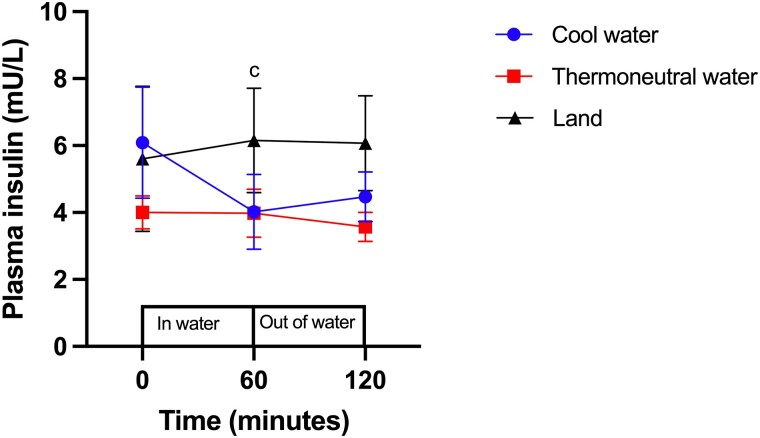
Effect of standing in cool water, thermoneutral water or on land on the concentration of plasma insulin (mU/L). c, indicates significant difference from baseline levels in cool water. Data are displayed as mean ± SEM (N = 7).

At time zero, the level of plasma insulin did not differ between the conditions (COOL vs THERMO, *P* = .31; COOL vs LAND, *P* = .86; THERMO vs LAND, *P* = .36; Fig. 2). There was no change in insulin level relative to baseline at 60 minutes of water immersion (t = 60 minutes; *P* = .31) or after 60 minutes of recovery (t = 120 minutes; *P* = .24) in THERMO. This was also the case after on-land standing (t = 60 minutes; *P* = .38) and recovery in LAND (t = 120 minutes; *P* = .48). At 60 minutes of water immersion in COOL, there had been a significant decrease in the concentration of plasma insulin from baseline, with a moderate effect size in the level of plasma insulin (*P* = .0015, Cohen d = 0.60; Fig. 2). Although not significant (*P* = .11; Fig. 2), there was a moderate effect size for the insulin level at 120 minutes of recovery compared to baseline in COOL (Cohen d = 0.51).

### Core Temperature

**Figure 3.**
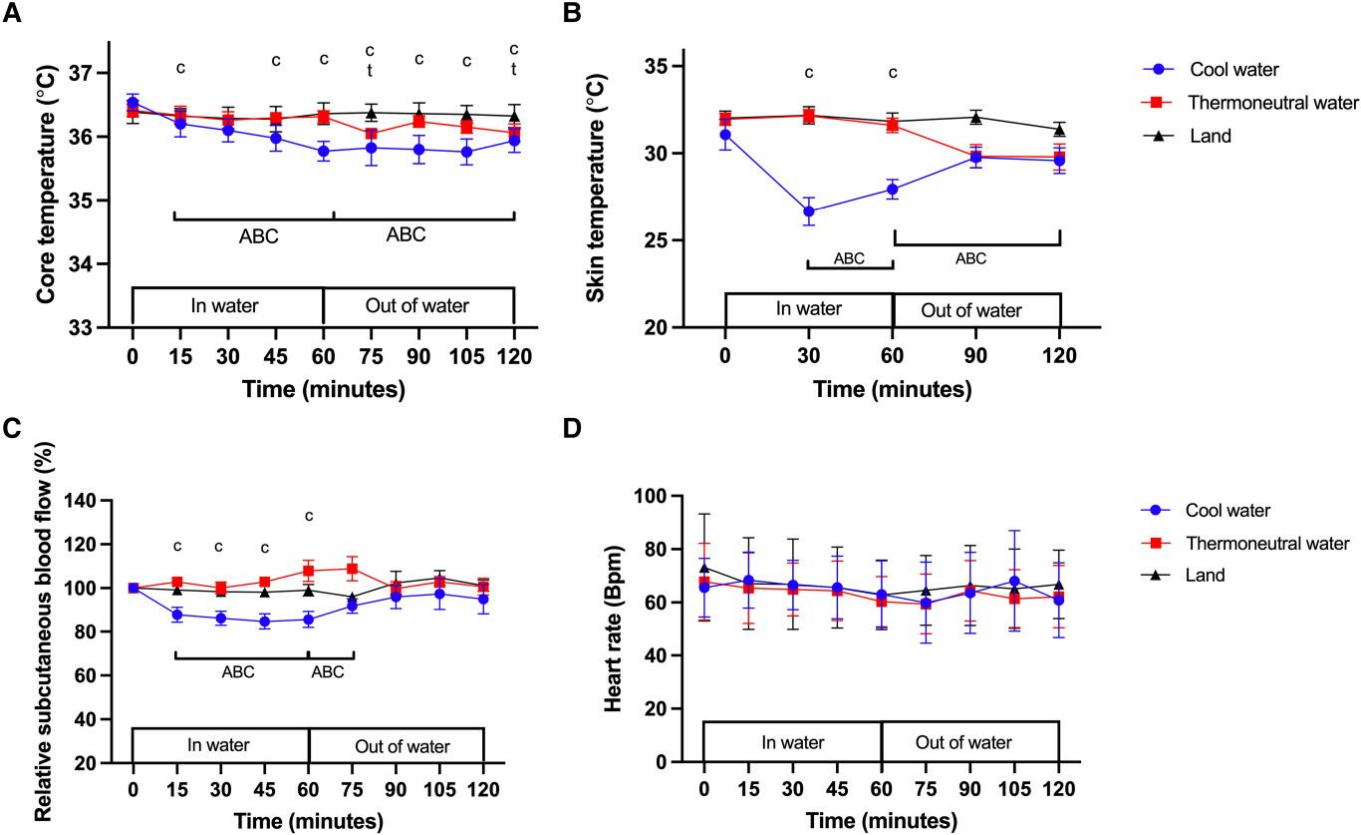
Effect of standing in cool water, thermoneutral water or on land on (A) core temperature, (B) mean skin temperature, (C) relative subcutaneous blood flow and (D) heart rate. c, significant difference from baseline in COOL; t, significantly different from baseline in THERMO. (A) For body temperature, the area between the baseline and curve (ABC) is significantly more negative in COOL vs THERMO and in COOL vs LAND during and after water immersion. (B) For mean skin temperature, the ABC is significantly more negative in COOL vs LAND and in COOL vs THERMO during water immersion. The ABC is also significantly more negative in COOL vs LAND and in THERMO vs LAND during recovery. (C) For relative subcutaneous blood flow, the ABC is significantly more negative in COOL vs THERMO and in COOL vs LAND during water immersion and early during recovery. Data are displayed as mean ± SEM (N = 8). (D) No significant changes in HR from baseline in COOL, THERMO or LAND during immersion or after recovery (*P* < .5).

At time zero there was no difference in core temperature between the experimental conditions (*P* > .05; Fig. 3A). Core temperature decreased by 0.8 ± 0.3 °C after 1 hour in COOL (*P* < .0001, Cohen d = 2.05) and remained low relative to the baseline, even at the end of the recovery period (t = 120 minutes; *P* = .011, Cohen d = 1.41). After 1 hour of immersion in THERMO, core temperature had not changed from baseline (*P* = .26), but core temperature fell to below baseline at 75 (*P* = .01, Cohen d = 2.51) and 120 minutes (*P* = .01, Cohen d = 2.91) during recovery. No change in core temperature was observed during LAND (*P* = .96) or after recovery (t = 120 minutes) in LAND (*P* = .99). The area between the baseline and core temperature × time curve in COOL was significantly more negative than in THERMO during immersion (*P* < .0001, Cohen d = 2.78) and recovery (*P* < .013, Cohen d = 1.89). Similarly, the area between baseline and the core temperature × time curve in COOL was significantly more negative than in LAND during standing (*P* < .005, Cohen d = 2.09) and recovery (*P* < .007, Cohen d = 1.64).

### Mean Skin Temperature

At time zero, there was no difference in mean skin temperature between the experimental conditions (*P* > .05; Fig. 3B). After 30 minutes in COOL, there had been a significant decrease in skin temperature relative to baseline (*P* = .02, Cohen d = 2.53; Fig. 3B), and skin temperature remained lower throughout the immersion period (Fig. 3B; *P* = .01, Cohen d = 2.17). Within 30 minutes of exiting the water in COOL, skin temperature had returned to baseline (*P* = .39). In THERMO, there were no change in skin temperature relative to baseline during immersion (*P* = .91) or recovery (*P* = .08). In LAND, there was no change in skin temperature while standing (*P* = .96) and at 1 hour of recovery compared to baseline (*P* = .61). The area between the baseline and the skin temperature × time curve during the immersion period was significantly more negative in COOL than in THERMO (*P* < .0001, Cohen d = 1.77) and in LAND (*P* < .0001, Cohen d = 1.70). During early recovery, the area between the baseline and the skin temperature × time curve in COOL was more negative than in LAND (*P* < .002, Cohen d = 1.33) and was significantly more negative in THERMO than in LAND (*P* = .02, Cohen d = 1.26).

### Subcutaneous Blood Flow

During COOL there was a 20% ± 9% decrease in subcutaneous blood flow from baseline at 60 minutes in COOL (*P* = .03, Cohen d = 2.51; Fig. 3C), and subcutaneous blood flow returned to the baseline level early during recovery (*P* = .96). During THERMO, subcutaneous blood flow did not change during water immersion (t = 60, *P* = .51) or recovery (t = 120 minutes, *P* = .99) relative to baseline. During LAND, subcutaneous blood flow did not change during standing (*P* = .95) or recovery (*P* = .99). The area between the baseline and the subcutaneous blood flow × time curve in COOL was significantly more negative than it was during THERMO throughout water immersion (*P* < .0001, Cohen d = 3.56) and during recovery (*P* = .002, Cohen d = 3.41). Similarly, the area between the baseline and the subcutaneous blood flow × time curve in COOL was significantly more negative than it was in LAND during standing (*P* = .003, Cohen d = 3.13) and recovery (*P* = .014, Cohen d = 2.92).

### Heart Rate

At time zero, there was no difference in HR between the experimental conditions (*P* > .05; Fig. 3D). After 1 hour (t = 60 minutes), there was no significant difference in HR in COOL (*P* = .79), THERMO (*P* = .25), or LAND (*P* = .15) relative to baseline values (Fig. 3D). There was also no change in HR at the end of recovery (t = 120 minutes) in COOL (*P* = .49), THERMO (*P* = .59), or LAND (*P* = .61) relative to baseline.

### Involuntary EMG Activity of the Deltoid and Trapezius Muscles

**Figure 4.**
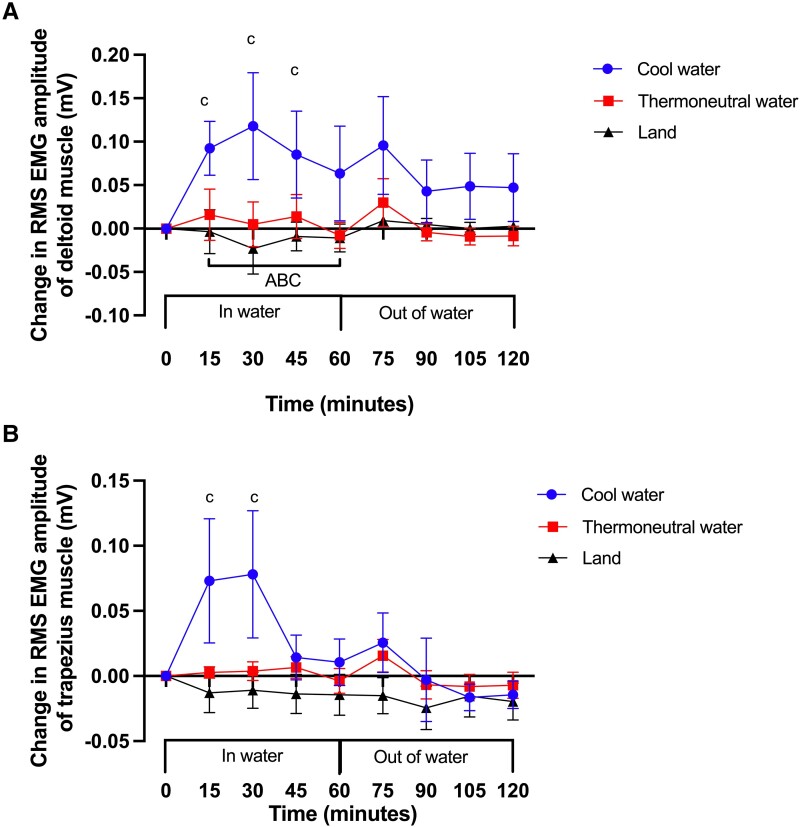
Effect of standing in cool water, thermoneutral water or on land on change in root mean square (RMS) of electromyography (EMG) amplitude of the (A) deltoid and (B) trapezius muscles expressed as millivolts (mV). c, significant difference from baseline in COOL. In both (A) and (B), the area between the baseline and curve (ABC) is significantly more positive in COOL vs LAND during water immersion. Data are displayed as mean ± SEM (N = 8).

Early during water immersion in COOL, the EMG activity of the deltoid muscle increased above baseline (t = 15 minutes, *P* = .02, Cohen d = 0.95; Fig. 4A), and remained elevated for up to 45 minutes (*P* = .03, Cohen d = 0.67), and then returned to baseline levels at 60 minutes (*P* = .19), and remained at baseline during the recovery period (t = 120 minutes, *P* = .48). There was no change in the EMG activity of the deltoid muscle from baseline during immersion (*P* = .99) or recovery in THERMO (*P* = .99). This was also the case in LAND during standing (*P* = .99) and recovery (*P* = .99). The area between the baseline and the EMG activity of the deltoid muscle vs time curve was significantly larger in COOL compared to LAND (*P* = .03, Cohen d = 1.27), and there was no difference between COOL and THERMO (*P* = .3).

Early during water immersion in COOL the EMG activity of the trapezius muscle increased above baseline (t = 15 minutes, *P* = .02, Cohen d = 0.95; Fig. 4B), remained elevated for about 30 minutes (*P* = .03, Cohen d = 0.67), returned to baseline levels at 45 minutes (*P* = .19), and remained at baseline during the recovery period (t = 120 minutes, *P* = .48). There was no change in EMG activity of the trapezius muscle from baseline during immersion (*P* = .99) or recovery in THERMO (*P* = .99). This was also the case in LAND during standing (*P* = .99) and recovery (*P* = .99). The area between baseline and the EMG activity of the trapezius muscle vs time curve was significantly higher in COOL compared to LAND (*P* = .03, Cohen d = 1.27), and there was no difference between COOL and THERMO (*P* = .12).

### Rate of Carbohydrate Oxidation

**Figure 5.**
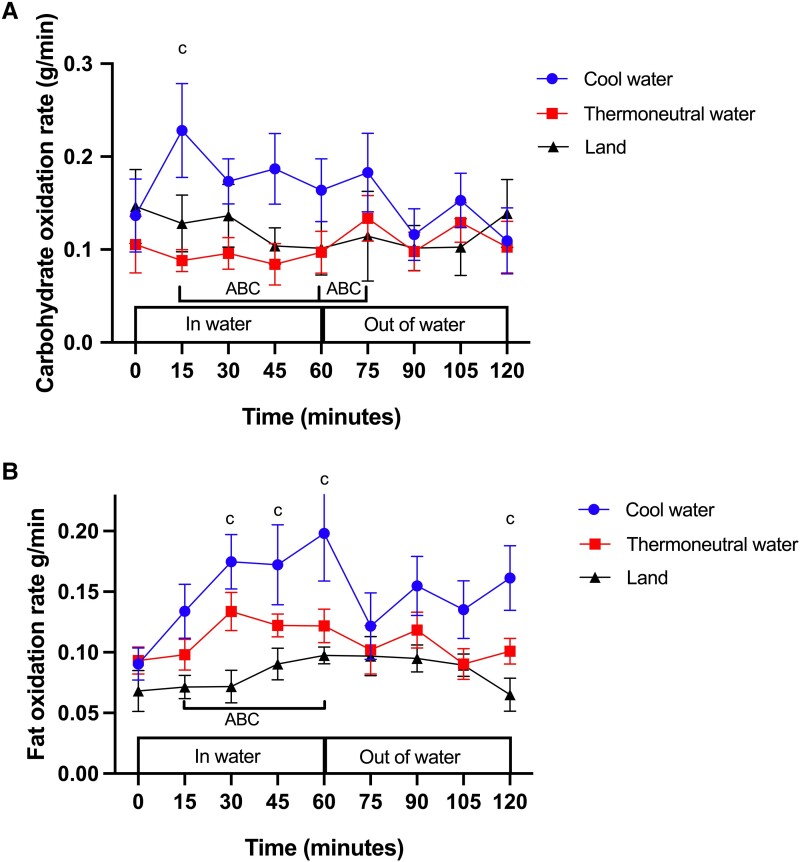
Effect of standing in cool water, thermoneutral water or on land on the rates of (A) carbohydrate and (B) fat oxidation. c, significantly different from baseline in COOL. (A) For carbohydrate oxidation, the area between the baseline and curve (ABC) is significantly more positive in COOL vs LAND during water immersion and early recovery. (B) For fat oxidation, the ABC is significantly more positive in COOL vs THERMO and in COOL vs LAND during water immersion. Data are displayed as mean ± SEM (N = 8).

At time zero, there was no difference in the rate of CHO oxidation between the experimental conditions (*P* > .05; Fig. 5A). During water immersion in COOL there was an almost 2-fold increase from baseline in the rate of CHO oxidation at 15 minutes (*P* = .005, Cohen d = 0.86). The rate of CHO oxidation then returned to baseline level (t = 30 minutes, *P* = .35) and remained at baseline throughout immersion (t = 60 minutes, *P* = .39) and recovery (t = 120 minutes, *P* = .40). In THERMO, the rate of CHO oxidation did not differ from baseline during (t = 60 minutes, *P* = .79) or after water immersion (t = 120 minutes, *P* = .93). Similarly, the rate of CHO oxidation during LAND did not change from baseline during standing (*P* = .16) or recovery (*P* = .81). The area between the baseline and the CHO oxidation rate × time curve in COOL was significantly higher during standing in LAND (*P* = .04, Cohen d = 1.11) and THERMO (*P* = .03, Cohen d = 1.15. Early during recovery, the area between the baseline and the CHO oxidation rate × time curve in COOL was significantly higher during standing in LAND (*P* = .034, Cohen d = 0.92), and there was no difference between COOL and THERMO (*P* = .2).

### Rate of Fat Oxidation

At time zero, there was no difference in the rate of fat oxidation between the experimental conditions (*P* > .05; Fig. 5B). During COOL, the rate of fat oxidation was higher at 30 minutes of water immersion (*P* = .02, Cohen d = 1.61) and remained higher than baseline until the end of the immersion in water (*P* = .02, Cohen d = 1.31) and was again higher at the end of recovery (*P* = .03, Cohen d = 1.21). In THERMO, the rate of fat oxidation did not change from baseline during water immersion (t = 60 minutes, *P* = .25) or recovery (*P* = .99). The rate of fat oxidation during LAND did not change compared to baseline during standing (*P* = .42) or recovery (*P* = .99). The area between the baseline and the fat oxidation rate × time curve was significantly higher during COOL than during THERMO (*P* = .03, Cohen d = 1.39) and standing in LAND (*P* = .04, Cohen d = 1.12).

## Discussion

Many people with T1DM participate in water-based activities at beaches where the water temperature is typically cool or cold [5]. We here show that immersion neck-deep in cool or thermoneutral water has no effect on the concentration of blood glucose in overnight fasted people with T1DM in a basal insulinemic state. However, recovery while on land after immersion in cool water caused a small increase in the concentration of blood glucose. Our findings suggest that this increase may result from a transient decrease in plasma insulin early after emerging from cool water. These low levels of insulin, in turn, probably result from the decrease in subcutaneous blood flow that occurred during immersion in cool water. On clinical grounds, our findings suggest that standing neck-deep in cool or thermoneutral water under conditions that mimic those typical of many beaches does not increase the risk of hypoglycemia during and early after water immersion in people with T1DM.

To the best of our knowledge, this is the first study that has investigated the response of blood glucose to standing in cool or thermoneutral water in people with T1DM, limiting our ability to compare our findings with those of past studies. The only study that has examined the effect of cold exposure on the concentration of blood glucose in T1DM is that of Bitton and colleagues [35]. Rather than immersing their participants in cool water, as we did, they found that 4 hours of localized application of cold water at the site of injection of slow-acting insulin in people with T1DM resulted in a decrease in the level of plasma insulin, as reported here, and a slow increase in the concentration of blood glucose. This latter finding differs from ours in that we found no change in blood glucose while our participants were immersed in cool water. Maybe the 1-hour exposure to cool water in our study was not long enough for blood glucose concentration to increase, which aligns with the finding of the aforementioned study [35]. Also, Bitton and colleagues [35] reported no further increase in blood glucose when their cooling stimulus was removed as opposed to the increase in blood glucose that was experienced by all our participants when they emerged from the cool water. These different results between studies may have to do with the fact that the removal of the local cool stimuli in Bitton et al [35] resulted in a quick return of insulin to baseline levels, whereas the insulin concentration in our study remained below baseline levels even after 60 minutes from emerging from the cool water. The observation that blood glucose is higher after breakfast on cold days than it is on warm days in insulin-treated people with T1DM [16] is consistent with the findings of Bitton et al [35], but not with our results. Of note, in people without T1DM, cold water immersion is associated with a small decrease in blood glucose (<0.5 mmol/L) in some studies [9, 12], whereas in other studies mild cold exposure causes no change in blood glucose [7, 8]. It is also important to note that none of the aforementioned studies concerned with whole-body exposure to cool or cold conditions [7-9, 12] tracked changes in the concentration of blood glucose when the cold stimulus was removed, thus preventing us from comparing our results post cool water exposure with those of others.

The absence of a decrease in the concentration of blood glucose during cool water immersion is somewhat surprising considering that this treatment was associated with an increase in the rate of CHO oxidation. The increase in shivering could explain, at least in part, the higher rate of CHO oxidation during immersion in cool water. Indeed, the higher EMG activity of the deltoid and trapezius muscles during immersion in cool water reveals that shivering was taking place, and a number of studies have shown that shivering increases the rate of CHO oxidation in people without diabetes [6-10]. Of note, although we did not directly measure the rate at which plasma glucose was oxidized, mild cold exposure on land (10 °C) or water (18 °C) has been shown to increase this rate by approximately 2-fold and to contribute to approximately 25% of whole-body CHO oxidation rate [7, 9]. On this basis, we propose that the increase in the rate of CHO oxidation reported here was at least partly due to an increase in the rate of plasma glucose oxidation.

The absence of a decrease in the concentration of blood glucose during immersion in cool water despite our proposed increase in the rate of plasma glucose oxidation can be explained on the grounds that cool water immersion was associated here with a significant decrease in the level of plasma insulin. The decrease in the level of plasma insulin was probably caused by the decrease in skin temperature and the accompanying decrease in subcutaneous blood flow reported here. A decrease in subcutaneous blood flow would decrease the rate of insulin release from its subcutaneous depot into the circulation. In support of this interpretation, Kølendorf and colleagues [15] found a strong correlation between subcutaneous blood flow and the rate of insulin absorption, with low skin temperature being associated with reduced subcutaneous blood flow [13, 14, 17]. Since a low level of plasma insulin would be expected to decrease the rate of peripheral glucose utilization and stimulate hepatic glucose production [36], it is possible that the decrease in plasma insulin could have led to an increase in the rate of hepatic glucose output that was high enough to match the higher rate of glucose oxidation that was occurring in the shivering muscle, thus resulting in a stable concentration of blood glucose. Also, since mild hypothermia in people without T1DM is associated with a decrease in insulin sensitivity [37], perhaps the mild hypothermia observed in the cool water condition caused a decrease in insulin sensitivity that further increased hepatic glucose production and opposed the increase in the rate of glucose oxidation. Further studies are required to assess if a decrease in insulin sensitivity in skeletal muscle also contributes to the increase in the concentration of blood glucose after immersion in cool water.

The progressive increase in the concentration of blood glucose during early recovery from cool water immersion can be explained by our finding that the rate of CHO oxidation decreased to baseline during recovery while the level of plasma insulin remained low. Assuming that changes in the rate of blood glucose oxidation reflect that of CHO oxidation, the decrease in the rate of CHO oxidation during recovery would be expected to be also accompanied by a decrease in the rate of blood glucose oxidation. This response together with the expected activation of hepatic glucose production by both the ongoing low level of insulin and hypothermia-mediated decrease in insulin sensitivity would altogether be expected to result in an increase in the concentration of blood glucose. Of course, in the absence of any measurement of the levels of counterregulatory hormones and of the rate of both endogenous glucose production and utilization, our interpretation remains to be tested.

The experimental conditions adopted for our study (warm or cool water as well as basal insulinemia) mimic those experienced by summer beachgoers before breakfast, a time of day when the risk of both hypoglycemia and exercise-mediated hypoglycemia are at their lowest in people with T1DM [38-40]. Our experimental conditions were also chosen to be relevant to the many individuals with T1DM who attend beaches with cool or warm water temperature. Indeed, the cool water condition adopted here (23 °C) falls within the range of water temperature of many beaches globally [4], making our findings relevant to people with T1DM residing in countries such as Australia, Brazil, California Croatia, Cyprus Greece, Malta, Mexico, and Spain. Although many individuals who go to the beach engage in various types of physical activities, a large proportion of beachgoers go to the beach solely to relax [41] and only stand relatively still or wade while in the water [19], in part because many are not confident swimmers and find waves hazardous [18]. Our findings are thus relevant to these individuals.

This study has some limitations. In particular, its relatively small sample size and the multiple comparisons performed for each outcome variable have the potential to increase the type 1 error rate. This potential limitation was addressed by comparing the area under the variable × time curve between conditions, in addition to comparing individual time points. Also, the probability of a type 1 error was highly unlikely to explain many of our findings given the very large Cohen d effect sizes and very low *P* values associated with these findings. Another limitation is that no isotope tracer techniques, muscle biopsies, or counterregulatory hormone assays were performed to elucidate the mechanisms underlying our findings and test the validity of our interpretations. Also, it is unclear whether similar findings would have arisen at lower temperatures that trigger more intense shivering. This is an important issue to address as higher-intensity shivering is associated with a greater reliance on the oxidation of plasma glucose [8, 42]. Unfortunately, the use of colder water was not an option. This was because the high level of discomfort associated with standing still for 1 hour in cold water would have made the recruitment of participants even more challenging than it was already for the current study. Also, the relevance of investigating the response of blood glucose to much colder water temperatures could be questioned, as people who go to the beach would be expected to avoid exposing themselves to very cold water unless they are wearing a dry or wet suit. Another limitation relates to the issue of whether similar findings would have been obtained from children with their large body surface to volume ratio, or in a demographic where the participants had higher adiposity. Another limitation stems from the omission of a non-T1DM control group, which limits our ability to compare our findings with individuals without T1DM. It is also unclear whether some of the variables examined here are affected by gender. Finally, since some individuals with T1DM in poor glycemic control have been shown to exhibit reduced cutaneous vasoconstriction and vasodilation in response to cold and heat stress [43], maybe their responses to cold water exposure would differ from those of our participants who were all complication-free.

Since all of the testing sessions were performed in the morning, after an overnight fast and under basal/near basal insulinemic conditions, this raises the issue of whether similar results would have been found had our study been performed at a different time of day, after an insulin bolus, or in a postprandial state. Also, and very importantly, since it is common for beachgoers to be active while they are in the water at the beach rather than standing still, as was the case here [44], further research should investigate how blood glucose in people with T1DM would respond to exercise in cool or cold water.

In conclusion, this study shows that standing in cool water at 23 °C or thermoneutral water while blood insulin is at a basal or near basal level does not increase the risk of hypoglycemia during and early after immersion in water. However, during the first hour following immersion in cool water, there was on average a small 2 mmol/L increase in the concentration of blood glucose that may have to be considered to achieve optimal blood glucose management.

## Data Availability

Some or all datasets generated during and/or analyzed during the current study are not publicly available but are available from the corresponding author on reasonable request.

## Abbreviations

ABC: area between the baseline and curve
CHO: carbohydrate
EMG: electromyography
HR: heart rate
SEM: standard error of the mean
T1DM: type 1 diabetes mellitus
